# Evaluating the performance of Plasmodium falciparum genetics for inferring National Malaria Control Program reported incidence in Senegal

**DOI:** 10.21203/rs.3.rs-3516287/v1

**Published:** 2023-11-01

**Authors:** Wesley Wong, Stephen F. Schaffner, Julie Thwing, Mame Cheikh Seck, Jules Gomis, Younouss Diedhiou, Ngayo Sy, Medoune Ndiop, Fatou Ba, Ibrahima Diallo, Doudou Sene, Mamadou Alpha Diallo, Yaye Die Ndiaye, Mouhamad Sy, Aita Sene, Djiby Sow, Baba Dieye, Abdoulaye Tine, Jessica Ribado, Joshua Suresh, Albert Lee, Katherine E. Battle, Joshua L Proctor, Caitlin A Bever, Bronwyn MacInnis, Daouda Ndiaye, Daniel L. Hartl, Dyann F Wirth, Sarah K Volkman

**Affiliations:** Harvard T. H. Chan School of Public Health; The Broad Institute; Centers for Disease Control and Prevention; Centre International de recherche, de formation en Genomique Appliquee et de Surveillance Sanitaire (CIGASS); Centre International de recherche, de formation en Genomique Appliquee et de Surveillance Sanitaire (CIGASS); Centre International de recherche, de formation en Genomique Appliquee et de Surveillance Sanitaire (CIGASS); Section de Lutte Anti-Parasitaire (SLAP) Clinic; Programme National de Lutte Contre le Paludisme; Programme National de Lutte Contre le Paludisme; Centre International de recherche, de formation en Genomique Appliquee et de Surveillance Sanitaire (CIGASS); Programme National de Lutte Contre le Paludisme; Centre International de recherche, de formation en Genomique Appliquee et de Surveillance Sanitaire (CIGASS); Centre International de recherche, de formation en Genomique Appliquee et de Surveillance Sanitaire (CIGASS); Centre International de recherche, de formation en Genomique Appliquee et de Surveillance Sanitaire (CIGASS); Centre International de recherche, de formation en Genomique Appliquee et de Surveillance Sanitaire (CIGASS); Centre International de recherche, de formation en Genomique Appliquee et de Surveillance Sanitaire (CIGASS); Centre International de recherche, de formation en Genomique Appliquee et de Surveillance Sanitaire (CIGASS); Centre International de recherche, de formation en Genomique Appliquee et de Surveillance Sanitaire (CIGASS); Institute for Disease Modeling, Bill and Melinda Gates Foundation; Institute for Disease Modeling, Bill and Melinda Gates Foundation; Institute for Disease Modeling, Bill and Melinda Gates Foundation; Institute for Disease Modeling, Bill and Melinda Gates Foundation; Institute for Disease Modeling, Bill and Melinda Gates Foundation; Institute for Disease Modeling, Bill and Melinda Gates Foundation; The Broad Institute; Centre International de recherche, de formation en Genomique Appliquee et de Surveillance Sanitaire (CIGASS); Harvard University; Harvard T. H. Chan School of Public Health; Harvard T. H. Chan School of Public Health

## Abstract

Genetic surveillance of the *Plasmodium falciparum* parasite shows great promise for helping National Malaria Control Programs (NMCPs) assess parasite transmission. Genetic metrics such as the frequency of polygenomic (multiple strain) infections, genetic clones, and the complexity of infection (COI, number of strains per infection) are correlated with transmission intensity. However, despite these correlations, it is unclear whether genetic metrics alone are sufficient to estimate clinical incidence. Here, we examined parasites from 3,147 clinical infections sampled between the years 2012–2020 through passive case detection (PCD) across 16 clinic sites spread throughout Senegal. Samples were genotyped with a 24 single nucleotide polymorphism (SNP) molecular barcode that detects parasite strains, distinguishes polygenomic (multiple strain) from monogenomic (single strain) infections, and identifies clonal infections. To determine whether genetic signals can predict incidence, we constructed a series of Poisson generalized linear mixed-effects models to predict the incidence level at each clinical site from a set of genetic metrics designed to measure parasite clonality, superinfection, and co-transmission rates. We compared the model-predicted incidence with the reported standard incidence data determined by the NMCP for each clinic and found that parasite genetic metrics generally correlated with reported incidence, with departures from expected values at very low annual incidence (<10/1000/annual [‰]). When transmission is greater than 10 cases per 1000 annual parasite incidence (annual incidence >10 ‰), parasite genetics can be used to accurately infer incidence and is consistent with superinfection-based hypotheses of malaria transmission. When transmission was <10 ‰, we found that many of the correlations between parasite genetics and incidence were reversed, which we hypothesize reflects the disproportionate impact of importation and focal transmission on parasite genetics when local transmission levels are low.

## Introduction

Pathogen genomics is revolutionizing public health by providing a rich data source for informing real-time, actionable recommendations for public health programs^1^. Each pathogen genome is a unique record of its previous transmission history that can be used to study the origin and spread of infectious diseases in real time. Genetic surveillance of pathogen populations provides an opportunity to characterize pathogen transmission structure and provide data-informed recommendations to public health programs to decrease transmission. In the past two decades alone, breakthroughs in genomic technologies and analytical techniques ^2–4^ have expanded pathogen genetic surveillance to a wide variety of viral and bacterial pathogens. Recent examples include the SARS-CoV-2 pandemic^5,6^, the 2013–2016 West African Ebola outbreaks^7,8^, and the Middle East Respiratory Syndrome (MERS) outbreaks in the Middle East^9^.

Despite this success, extending genetic surveillance to more complex pathogens, such as the eukaryotic *Plasmodium falciparum* parasite that is the causative agent for the deadliest form of malaria, has been challenging. Unlike viral or bacterial pathogens, *P. falciparum* has a complex, 23-megabase genome with over 5000 genes whose genomic architecture is heavily influenced by meiotic recombination^10^. *P. falciparum* must undergo sexual reproduction within a mosquito vector to complete its life cycle prior to being transmitted to a new human host. The sexual nature of the *P. falciparum* parasite complicates many of the phylogenetic and phylodynamic techniques used in viral and bacterial genetic surveillance studies ^2–4^.

Malaria genetic surveillance has instead relied on identifying genetic epidemiology metrics that summarize the changes in parasite genetics observed from the empirical sampling of parasite genomes from malaria endemic regions. These genetic epidemiology metrics include the frequency of multiple strain (polygenomic) infections^11,12^, the number of strains per infection (complexity of infection, COI), the genetic relatedness of parasite strains^13,14^, and the frequency of clonal parasites in the population^15,16^.

Many of these genomic epidemiology metrics were identified by comparing sites with different levels of transmission intensity, whose measurement includes prevalence (frequency of infections), incidence (rate of new infections), and the entomological inoculation rate (EIR, number of infectious mosquito bites per individual). As such, malaria genetic epidemiology metrics tend to be associated with transmission intensity. Regions with high transmission intensity are expected to have high frequencies of polygenomic infections and high COIs because individuals are more likely to be exposed to multiple infectious bites^17^ and there is greater opportunity for parasite outcrossing. Conversely, regions with low transmission intensity are expected to have high frequencies of clonal or genetically related parasites^18,19^ due to increased levels of inbreeding associated with declining transmission and smaller effective parasite population sizes. However, the exact relationship between these genetic metrics and epidemiological measures of transmission intensity is unknown, and it is unclear whether these relationships are consistent across the range of transmission from low to high intensity. It is also unclear to what extent other epidemiological factors, such as transmission heterogeneity (e.g., focal transmission) and importation, affect these genetic epidemiology metrics.

In this study, we focused on studying the relationship between parasite genetics and malaria incidence as reported by the National Malaria Control Program (NMCP). Malaria transmission in Senegal is highly heterogeneous and dependent on geographic location, ranging from < 1‰ to > 1000‰ annual incidence. This geographic disparity was ideal for evaluating the relationship between parasite genetics and incidence across a range of transmission intensities in a limited geographic area where reported incidence and genetic epidemiology metrics could be measured consistently across study sites and years. We used a series of mathematical models to quantify the relationship between parasite genetics and incidence and identify transmission regimes (regions within the incidence parameter space) where the relationships between parasite genetics and incidence differ. Identifying these transmission regimes is important because they can arise from fundamental changes in transmission structure that affect how parasite genetics can be used to study transmission and develop data-informed public health recommendations.

## Results

### Study Design Overview

**Figure 1:**
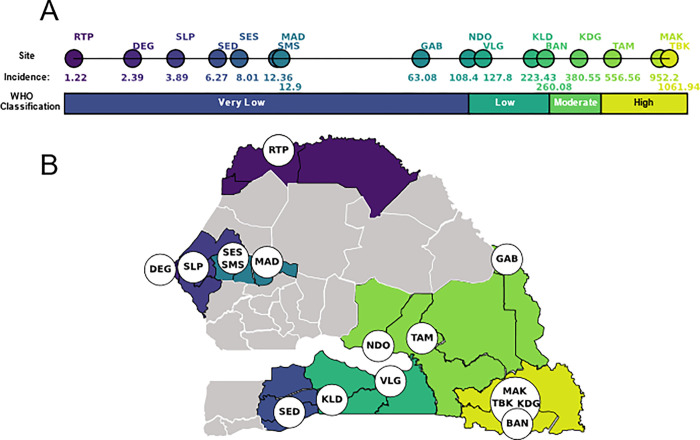
Study Design Overview. Samples were collected from 16 sites throughout Senegal. (**A**) The average, NMCP-reported annual incidence values (‰) for each site during the time of sampling and their transmission classification according to the WHO (**B**) The locations of each of the sampling sites.

We genotyped 3,147 *P. falciparum* clinical infections collected using PCD at 16 health facility clinic sites in Senegal with a 24 SNP molecular barcode^20^ (Figure 1, **Supplemental Figure 1**). Five genetic metrics (polygenomic fraction, COI, monogenomic clone proportion, R_H_^13^, and the cotransmission fraction) were calculated from the molecular barcode data for each site-year (Table 1, **Supplemental Figure 2**). Polygenomic fraction, COI, and R_H_ were expected to be positively correlated with incidence, while monogenomic clone proportion and the cotransmission fraction were expected to be negatively correlated with incidence (Table 1).

Malaria transmission in Senegal is highly heterogeneous, and the annual incidences reported by the NMCP for the clinic sites ranged from < 1 to >1000 ‰ (Figure 1A). Based on the definitions established by the WHO^21,22^, eight sites were “very low” transmission settings (< 100 ‰), three were “low” transmission settings (100–250 ‰), two were “moderate” transmission settings (250–450 ‰), and three were “high” transmission settings (>450 ‰) (Figure 1A). Five sites were sampled for multiple years: Dalaba (KDG, 2015–2020), Touba (MAD, 2019–2020), Sessene (SES, 2018–2020), Thies (SLP, 2015–2020), and Richard Toll (RTP, 2012–2015). The remaining 11 sites were sampled in either 2019 or 2020 or both (Table 2). NMCP estimates of annual incidence were calibrated to the clinic catchment site.

### Transmission rank analyses reveal limitations of using individual genetic metrics to infer incidence

**Figure 2:**
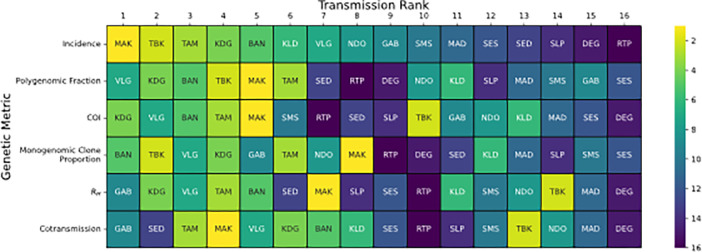
**Rank Analysis for each sample site based on the genetic metrics** used in this study is shown. Transmission rank is defined as the rank one would use when using the metric to infer transmission intensity (Table 1), with 1 being the highest and 16 being the lowest. Assigned transmission ranks were assigned based on the average value calculated across sample years. For polygenomic fraction (**A**), *The REALMcCOI* COI (**B**), and R_H_ (**C**), the transmission rank is positively correlated with the metric. For cotransmission fraction (**D**) and monogenomic clone proportion (**E**), the transmission rank is negatively correlated with the metric. The solid black line between transmission rank 7 and 8 indicates the threshold used when grouping transmission ranks into a 2 × 2 contingency table.

We first evaluated whether parasite genetics could be used to reliably rank sites by transmission intensity by assigning each of the 16 sites examined in this study a transmission rank based on the expectations^23–25^ listed in Table 1 and comparing them with the rank assigned by incidence (Fig 2). This approach was designed to mimic inferences made using data collected from a single parasite genetic metric. A major goal of this rank analysis was to evaluate the consistency of the transmission ranks assigned by each genetic metric. For sites with multiple years, the rank was based on the average value. Overall, the consistency between the two was weak, with small to moderate amounts of concordance (Kendall rank correlation coefficient < 0.38, **Supplemental Table 1**). Of the genetic metrics examined, only the rank correlation between monogenomic clone proportion and incidence was statistically significant (Kendall rank correlation = 0.38, *p*-value = 0.04).

However, transmission ranks tended to be consistent when grouping the data into high and low transmission sites. Transmission ranks were organized into 2 ´ 2 contingency tables (incidence *versus* each of the genetic metrics) with two categories: high transmission ranking (transmission rank < 8) and low transmission ranking (transmission rank ≥ 8). The threshold at 8 was determined by visual analysis of the transmission rank switching and those with a transmission rank ≥ 8 correspond to the WHO-defined categorization of “very low” transmission. Splitting the transmission rank data improved the correlation between each of the genetic metrics and incidence (Yule’s *Q* between 0.47 and 0.96, **Supplemental Table 1**). Monogenomic clone proportion, polygenomic fraction, and cotransmission fraction were correlated with these incidence groupings, but only monogenomic clone proportion was statistically significant (Yule’s *Q* = 0.96, Pearson’s chi-square correlation *p*-value = 0.01).

### Multivariate regression analyses identify different correlations between parasite genetics and NMCP-reported incidence

The transmission rank analyses showed that, while some genetic metrics could be used to broadly differentiate sites with annual incidence greater versus less than < 100 ‰ , the exact transmission rank assigned by each genetic metric conflicted with one another and with incidence. One way of resolving these conflicts would be to use a multi-variate regression model to generate a single, site-specific incidence prediction using all the information collected from each genetic metric. We constructed a series of Poisson mixed effects generalized linear models (GLM, Methods) that quantify the relationship between genetics and incidence based on different combinations of the five genetic metrics used in this study. This approach allowed us to evaluate the predictive power of each genetic metric alone and in combination with one another.

**Figure 3:**
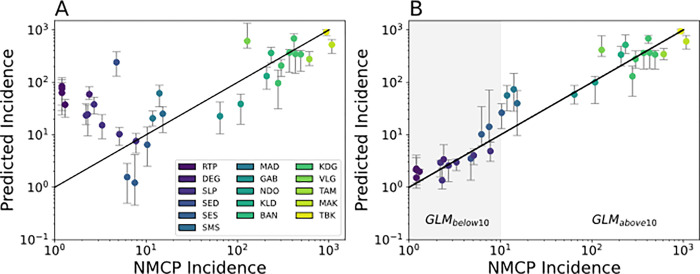
**Poisson mixed effect GLM model predictions** based on all five genetic metrics used in this study using (**A**) all the site-years and (**B**) from a piecewise GLM that splits the data in to low (GLM_below10_, shaded in grey) and high transmission sites (GLM_above10_). Each dot is the predicted incidence for all the examined site-years. Error bars represent the 95% confidence interval generated through iterative leave one out analyses where all the site-years associated with a chosen site were left out of the analysis. The legend applies to both **A** and **B**.

Model predictions from a GLM utilizing all five genetic metrics revealed two parameter regions with opposing model bias corresponding to regions with annual incidence > 10 ‰ and those with < 10 ‰. (Figure 3). Overall, model predictions for regions with annual incidence > 10 ‰ were consistent with the reported data (Figure 3A). However, the model tended to underestimate incidence relative to the official, NMCP-reported incidence values (**Supplemental Figure 3A**). When annual incidence is > 10 ‰, increasing incidence was associated with increasing polygenomic fraction (Pearson correlation coefficient *r* = 0.77, *p*-value = 1.6e-4) and COI (*r* = 0.60, *p*-value = 8.6e-3), but decreasing monogenomic clone proportion (*r* = 0.60, *p*-value = 9.8e-6) (**Supplemental Figure 4**). R_H_ and cotransmission fraction were also negatively associated but their correlations were not statistically significant (*r* = −0.27, *p*-value = 0.29 and *r* = −0.17, *p*-value = 0.496, respectively).

**Figure 4:**
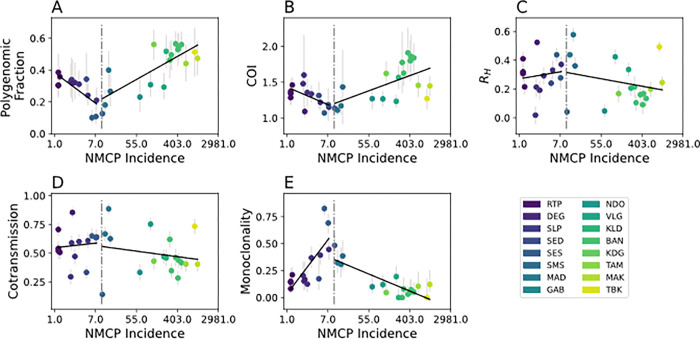
Relationship between incidence and (**A**) polygenomic fraction, (**B**) *THE REAL McCOIL* COI, (**C**) R_H_, (**D**) cotransmission fraction, and (**E**) monogenomic clone proportion. Error bars represent the 95% confidence interval for each examined site-year. The black lines represent model ordinary least squares regression model fits. A vertical dotted line is drawn at an NMCP-reported incidence of 10 ‰.

The patterns observed in regions with annual incidence < 10 ‰ differed from those observed in regions with > 10 ‰. Unlike in the higher transmission regions, the model predictions were not consistent with the NMCP-reported incidences and were consistently overestimated. The inability of the model to accurately predict incidence in regions with < 10 ‰ was because many of the correlations between parasite genetics and incidence differed from those observed in higher transmission regions (Figure 4). When annual incidence < 10 ‰, sites with higher incidence were associated with decreasing polygenomic fraction (*r* = −0.75, *p*-value = 2.0e-3) and COI (*r* = −0.60, *p*-value = 0.04), but increasing monogenomic clone proportion (*r* = 0.77, p-value =1.38e-3). The correlations between R_H_ and cotransmission fraction were also reversed but statistically insignificant (*r* = 0.10, *p*-value = 0.73 and *r* = 0.12, *p*-value = 0.69, respectively).

Model predictions were also made using downsampled estimates of the monogenomic clone proportion to control for differences in monogenomic infection sample size (**Supplemental Figure 5**). Downsampled monogenomic clone proportion estimates were lower (**Supplemental Figure 5A-B**) but otherwise had no major effect on model predictions (**Supplemental Figure 5C**).

Based on these observations, we generated a piecewise GLM model that splits the data into two groups, one for annual incidence < 10 ‰ (GLM_below10_) and one for annual incidence > 10 ‰ (GLM_above10_). The inflection point that separates GLM_below10_ and GLM_above10_ was determined by performing a parameter sweep over different incidence values (**Supplemental Figure 6**). However, our dataset had a noticeable gap in sampling for sites with annual incidences between 10–100 ‰, which made it unclear how reliably we could identify the inflection point. For the purposes of this study, we used a conservative threshold of 10 ‰ because it represents the minimum value where splitting the data caused the model fits to improve. The resulting piecewise model significantly improved model fits (AIC [Akaike Information Criterion] = 1291.34) relative to the original GLM (AIC = 3065.78), but tended to overestimate incidence when the reported values were close to the inflection point at 10 ‰ (Figure 3B, **Supplemental Figure 3B**).

### Parasite genetics can be used to estimate incidence in low to high transmission settings

**Figure 5:**
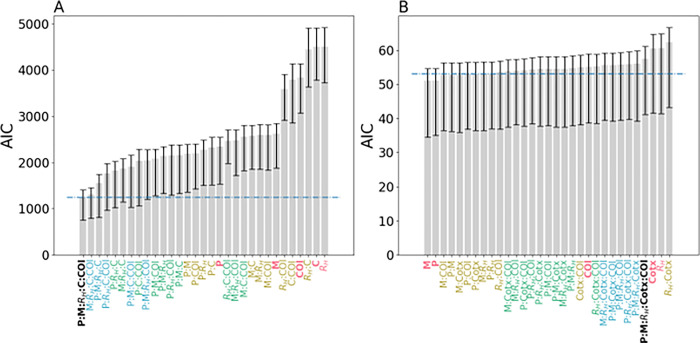
AIC values for (**A**) GLM_high_ and (**B**) GLM_low_ models calibrated with different combinations of genetic epidemiology metrics. P refers to polygenomic fraction, Cotx refers to cotransmission fraction, COI refers to *THE REAL McCOIL* COI, and M refers to monogenomic clone proportion. For convenience, models trained with all five genetic epidemiology metrics are labeled in black, four in blue, three in green, two in yellow, and one in red. Error bars indicate the 95% confidence interval obtained from leave-one-out cross-validation. The dotted blue line indicates the line of statistical significance relative to the best fitting model. Bars whose average is above the dotted line are statistically worse than the best-fitting model. Bars whose average is below the dotted line perform equally well as the best-fitting model.

We next evaluated whether each genetic metric was sufficiently powered to estimate incidence by itself or whether there was a minimum subset of genetic metrics that could be used to accurately estimate incidence. Because the piecewise GLM model strongly suggests that parasite genetics reflect different epidemiological dynamics when annual incidence < 10 ‰, we assessed whether the predictive power of these genetic epidemiology metrics was the same in regions with annual incidence < 10 ‰ and those with > 10 ‰. To answer these questions, we generated a series of GLM_below10_ and GLM_above10_ models (collectively referred to as {GLM_below10_} and {GLM_above10_}) that were trained with different combination of genetic epidemiology metrics and compared their relative goodness-of-fits using AIC (Figure 5). The goal was to identify the set of genetic epidemiology metrics that resulted in the best-fitting model for both {GLM_below10_} and {GLM_above10_}.

For the {GLM_above10_} models (Figure 5A), the best fitting model was the one that included all five genetic metrics (GLM_above10_, AIC = 1245.86). When examining each genetic metric individually, the polygenomic fraction was the best predictor of incidence (AIC = 2342.57), followed by the monogenomic clone proportion (AIC = 2624.32) (**Supplemental Figure 7**). R_H_ and cotransmission were the worst individual predictors (AIC = 4501.90 and 4501.93 respectively) but including them in combination with other metrics improved model fit. In general, increasing the number of genetic metrics improved model fit. The average AIC for models with one, two, three, and four genetic metrics was 3560.88, 2858.25, 2178.32, and 1719.56 respectively.

For the {GLM_below10_} models, there was no single, statistically best fitting model (Figure 5B). The model with the lowest AIC was the one trained on monclonality alone (AIC = 51.05), followed by the one trained on poylgenomic fraction alone (AIC = 51.16). However, based on standard definitions used to determine statistical significance (AIC difference > 2), it was not possible to determine which of the eight models with AIC < 53.05 was the best. On average, including additional genetic metrics did not improve model fit. The average AIC for models with one, two, three, and four genetic metrics was 55.68, 54.10, 54.44, and 55.86. The AIC for GLM_below10_, the original model trained on all five genetic metrics, was 57.52.

### Extrapolation of parasite genetic epidemiology based on trends seen in transmission settings with annual incidence > 10 ‰ overestimates transmission in transmission settings with annual incidence < 10 ‰

Based on these results, we next evaluated whether inappropriately applying the trends in parasite genetics observed in moderate-to-high transmission settings would cause erroneous incidence predictions in very low transmission settings. To test this, we used GLM_above10_ to generate incidence predictions for the sites with annual incidence < 10 ‰ (**Supplemental Figure 8**). This approach reflects the current state of malaria genomic epidemiology analyses, where incidence is inferred based on the expectations stated in Table 2. The incidence values predicted by GLM_above10_ were similar (**Supplemental Figure 8A**) to those seen from the GLM that was trained on all the data (Figure 3A) and settings with annual incidence < 10 ‰ were consistently overestimated.

## Discussion

Parasite genetics has the potential to enable public health officials to evaluate changes in transmission in settings where the corresponding epidemiological data are either missing or difficult to collect. However, the utility of parasite genetic surveillance will depend on how informative genetics is for studying malaria transmission and whether the inclusion of genetics can enhance the confidence of estimates based on standard epidemiological measures of transmission. The major goals of this study were to (1) characterize the relationship between five malaria genetic epidemiology metrics and incidence to determine whether these relationships were constant across transmission strata, and (2) test the predictive power of five malaria genetic epidemiology metrics for inferring transmission intensity, which in this study was measured as the NMCP-reported incidence for the catchment health facility.

Senegal was an ideal setting for this analysis due to its extensive range of transmission intensities in a localized geographic region. By utilizing data collected across 16 health facilities located throughout the country, we found that the relationship between parasite genetics and annual incidence changed in very low transmission settings with < 10‰. Based on these results, we recommend that parasite genetics be used to evaluate changes in incidence in when the annual incidence > 10‰ and used to assess potential sources of importation and other forms of transmission heterogeneity when transmission is low and falls below an annual incidence of 10‰.

When transmission is above an annual incidence > 10‰, the relationship between parasite genetics and reported incidence were consistent with previously established superinfection-based hypotheses that predict higher rates of multiple infections as transmission intensity increases^23–25^. Under these conditions, we show parasite genetics can be used to accurately infer incidence and that increasing transmission intensity is associated with an increase in polygenomic fraction and COI and a decrease in the frequency of clonal parasites in the population. These results suggest that national malaria control programs can utilize these correlations to quantify and compare the incidences of regions where transmission is high enough to be explained by superinfection, which in Senegal occurs when annual incidence > 10‰. Importantly, these inferences can be made with as few as 24 SNPs and approximately 50–100 samples per site without whole genome sequencing, which could be advantageous for assessing changing levels of transmission in high transmission settings with limited resources. These 24 SNPs can be genotyped from the genomic material extracted from discarded RDTs, which greatly reduces the technical and logistical complexities involved with collecting appropriate genetic material from clinical populations^26^. While estimates of incidence can be inferred from polygenomic fraction and monogenomic clone proportion alone, the most accurate inferences of incidence require utilizing multiple genetic metrics that collectively assess the impact of clonal transmission, superinfection, and cotransmission.

However, the relationship between parasite genetics and incidence was not consistent across all transmission strata. When transmission falls below annual incidence < 10‰, we found that many of the relationships between parasite genetics and incidence observed in higher transmission regions were reversed; increasing transmission intensity resulted in a decrease in polygenomic fraction and COI and an increase in the frequency of clonal parasites in the population. These results are difficult to explain under superinfection-based hypotheses, especially as the study sites with the lowest incidence, such as Richard Toll, had polygenomic fractions that were consistent with those seen in study sites with annual incidence> 400‰.

One possibility for the unusual trends in parasite genetics in very low transmission settings is that accurate quantification of the NMCP-reported incidence values is more difficult in as transmission declines because infected individuals are infrequent and difficult to identify. Superinfection in very low transmission settings may also be more difficult to detect as parasite populations become more clonal and genetically related^27^. While we cannot discount problems associated with sample ascertainment or measurement error in low transmission settings, it is difficult to attribute our observations to sample ascertainment bias alone given the tight, but reversed, correlation observed between polygenomic fraction, monogenomic clone proportion, and COI in this transmission regime.

Instead, we suppose that these changes are driven by fundamental changes in transmission structure that affects the parasite genetics of very low transmission settings^28^. In Senegal, we suspect the reversed relationships between parasite genetics and transmission intensity reflects the disproportionate impact of importation as local transmission declines. The 2013 Senegal census estimated that 14.6% of the population were internal lifetime migrants, meaning that their current area of residence differs from their birthplace^29^. The most popular destinations of internal lifetime migrants are in the low and very low transmission regions, such as Dakar, Diourbel, and Thies. Richard Toll also experiences seasonal influxes of migrant workers due to the presence of the Senegalese Sugar Company, and we previously showed that identical parasite clones could be detected between Dakar and Richard Toll^29^. Anecdotal evidence obtained from the health facilities in the low transmission regions of this study suggest that patients with recent travel history were more likely to be tested and diagnosed with malaria. Regions with moderate to high levels of transmission, such as Kaolack and Kedougou, reported a net loss in population in the 2013 census. Thus, while it is possible to infer incidence from parasite genetics in the very low transmission settings of Senegal (Fig. 3B, GLM_below10_), we suspect this is mainly driven by the importation of parasites from the moderate- to high-transmission regions to the lower transmission regions of Senegal. We recommend that the parasite genetics of very low transmission settings be combined with data regarding travel history or other indicators of human movement ^28,30^ to evaluate the potential impact of importation or focal transmission.

Overall, these results suggest that there are two distinct regimes where parasite genetics could be used to inform public health decision-making. When transmission is sufficiently high such that superinfection dominates, changes in parasite genetics can be used to infer incidence and quantify the transmission intensity in different regions. Parasite genetics could be especially valuable for evaluating the efficacy of public health interventions in reducing transmission in moderate to high transmission settings. However, when transmission falls below a certain threshold, our results suggest that parasite genetics should instead be used to begin evaluating the impact of importation or other heterogeneous transmission processes whose effects are masked by local mixing and transmission in high transmission settings but whose contributions are proportionally greater in low transmission settings. The exact incidence threshold for distinguishing between these two paradigms is uncertain, but likely lies between an annual incidence of 10 and 100‰. Until additional study sites with annual incidence between 10 and 100‰ can be examined, we suggest using the current WHO guidelines for defining sites with very low transmission sites (annual incidence < 100‰) to determine when parasite genetics can be used to infer transmission intensity and when it can be used to study subtler effects associated with importation and other sources of transmission heterogeneity.

The careful examination of parasite genetics in very low transmission sites could help national malaria control programs address long standing issues regarding the role of importation and focal transmission in low transmission settings. Very low transmission sites should be identified prior to using parasite genetics, as inappropriately applying the trends in parasite genetics from higher transmission settings risks over-estimating incidence and ignores possible sources of infections in low transmission areas. This phenomenon is most clearly seen in the model predictions from the GLM trained on all the sites (Fig. 3A) and the out-of-sample extrapolations made with the GLM trained on only sites with annual incidence > 10‰ (GLM_above10_, **Supplemental Fig. 8**). In practice, very low transmission sites can be distinguished from higher transmission sites using standard epidemiological metrics of incidence. The advantage of parasite genetics in very low transmission settings is that it could potentially allow national malaria control programs to identify source-sink populations or focal transmission sites that require targeted intervention for elimination. Additionally, parasite genetics in very low transmission sites could potentially be used to help countries confirm the absence of locally sustained transmission when applying for WHO certification of elimination.

In conclusion, we evaluated the relationship between several parasite genetic epidemiology metrics and transmission intensity measured as annual incidence in Senegal. Our results clearly show that parasite genetic metrics behave differently under diverse transmission strata. When transmission is sufficiently high, changes in parasite genetics were consistent with different rates of superinfection and outcrossing as transmission intensity changes. However, when transmission intensity falls too low, changes in parasite genetics cannot be explained by current superinfection-based hypotheses; we instead hypothesize that they represent importation or other forms of transmission heterogeneity. These results highlight the multidimensionality of parasite transmission and demonstrate the utility and limitations of parasite genetics for inferring transmission intensity in Senegal. Future studies will continue to investigate the relationship between parasite genetics and other epidemiological metrics of transmission and importation as well as incorporate other genetic epidemiology metrics that were not explored in this study (*e.g*., clonal barcode persistence^14,31^).

## Methods

### Sampling strategy

Samples from health facilities were collected as previously described^13^. Samples were collected through PCD from febrile patients reporting to health posts or clinics during the malaria transmission season in Senegal (September to December), or actively detected in households in response to a case detected at the Richard Toll clinics. Patients over 6 months of age presenting to the clinic with fever within the past 24 hours and no history of antimalarial use were diagnosed with malaria using microscopy or rapid diagnostic tests (RDTs). Parasite genomic DNA was extracted from filter papers spotted with blood samples collected from malaria-positive patients at all sites except Richard Toll. For Richard Toll, parasite genomic DNA was extracted from malaria positive RDT cassettes.

Ethical approval for these studies was obtained from the Ministry of Health and Social Action in Senegal (Avis Protocol SEN1949) and the Harvard T.H. Chan School of Public Health Institutional Review Board (Protocol 16330).

### Barcoding with a 24 SNP molecular barcode

The 24 SNP molecular barcode^20^ provides a high-level snapshot of genetic diversity that trades genomic resolution for epidemiological sampling breadth. The barcode consists of 24 neutral SNPs spread throughout the malaria genome that are genotyped using a panel of TaqMan-based quantitative PCR genotyping assays. Nucleic acid material was extracted from either filter paper or RDT and preamplified using previously established methods^26,32^. The criteria for calling homozygous and heterozygous sites were based on those described previously^13^.

### Parasite genetic epidemiology quantification

Each of the five genetic epidemiology metrics were calculated for each site-year according to previously established methods. Polygenomic infections were identified as those infections whose barcode had two or more heterozygous sites. Polygenomic fraction was calculated by dividing the number of polygenomic infections by the total number of samples collected. Monogenomic clone proportion was defined as the proportion of monogenomic infections infected with a barcode genotype that is present in more than one infection in the population. It was calculated as $monoclonality=1-p_{\mathrm{mono,unique}}$, where $p_{\mathrm{mono,unique}}$ is the proportion of monogenomic infections infected with a unique barcode genotype. R_H_ and the cotransmission fraction were calculated as previously described^13^. *THE REAL McCOIL*^33^ COI was calculated independently for each study site using the categorical method with the following parameter values: maxCOI = 25, threshold_ind = 20, threshold_site = 20, and err_method = 3. All other parameters used the default values. The median value estimated by *THE REAL McCOIL* was used as the point estimate of COI for each sample.

### NMCP-reported Incidences

When possible, we used the NMCP-reported, district-level incidences for each collection site. However, this data was only readily available for the data collected in and after 2019. For older data, we used the region-level incidences reported in the annual NMCP reports^34–40^.

### Poisson Generalized Linear Model

Model predictions were made with a Poisson Generalized Linear Model:

1 $$log\left(\lambda_{i}\right)∼x_{i}^{T}\beta$$

where $\lambda_{i}$ is the predicted incidence for a given site-year, $x_{i}$ is the vector containing the values of the covariates used in the model, and β is the vector of coefficients to be estimated. $x_{i}$ includes the polygenomic fraction, monogenomic clone proportion, R_H_, COI, and cotransmission fraction for each site-year and two categorical variables for (1) the region of origin and (2) whether incidences were measured at the district-level or at actual health facility catchment area. Models were fit using the GLM function in the Python 3 package *statsmodel* (v0.13.5).

Leave-one-out cross-validation was performed by splitting the dataset by sampling site and using all but one of the sites during model fitting. All site-years associated with the chosen site were removed from model fitting and was repeated for each of the 16 studied sampling sites. The estimates reported in this study used the average value obtained from leave-one-out cross-validation.

### Akaike Information Criterion

AIC was calculated as:

2 AIC=2k-2L

where k is the number of free parameters and L is the log likelihood. For the GLM trained on all the data, k=7 (one for each of the parameters used in the GLM). For the piecewise GLM, k=8, to include the additional incidence threshold parameter that separates GLM_below10_ from GLM_above10_.

## Figures and Tables

**Table 1.** Summary of the genetic metrics used in this study and their expected relationship with transmission intensity. Clones are defined as a parasite whose barcode is observed more than once in the population and is calculated only for monogenomic infections. COI was calculated using *THE REAL McCOIL*.

Genetic Metric	Definition	Transmisison Reflected	Expectation
Polygenomic Fraction	Proportion of infections that are polygenomic	Superinfection & Cotransmission	Increase with transmission intensity
Complexity of Infection (COI)	Number of strains per infection	Superinfection & Cotransmission	Increase with transmission intensity
Monogenomic clone proportion	Proportion of monogenomic infections carrying a clone that was sampled elsewhere in the population	Clonal Transmission	Decrease with transmission intensity
R_H_	Estimate of polygenomic inbreeding based on intra-host heterozygosity	Superinfection & Cotransmission	Decrease with transmission intensity
Cotransmission Fraction	Proportion of polygenomics resulting from cotransmission	Cotransmission	Decrease with transmission intensity

**Table 2.** The regions, district, three-letter code, and sampling years for each site. Sites with ACD of household communities are asterisked.

REGION (RM)	DISTRICT (DS)	SITE (PS)	CODE	SAMPLE YEARS
Kedougou	Kedougou	Tomboronkoto	TBK	2019
Kedougou	Kedougou	Mako	MAK	2019
Tambacounda	Tambacounda	Dialocoto	TAM	2019
Kedougou	Kedougou	Dalaba	KDG	2015–2020
Kedougou	Kedougou	Bandafassi	BAN*	2019
Kolda	Kolda	Bagadadji	KLD	2019
Kolda	Velingara	Ouassadou	VLG	2019
Tambacounda	Makacoulibantang	Ndoga Babacar	NDO*	2019
Tambacounda	Bakel	Gabou	GAB	2019
Diourbel	Diourbel	Madiyana 2	MAD	2019–2020
Diourbel	Diourbel	Keur Serigne Mbaye Sarr	SMS	2019
Diourbel	Diourbel	Sessene	SES*	2018–2020
Sedhiou	Sedhiou	Centre De Santé Goudomp	SED	2019
Thies	Thies	SLAP (Service de Lutte Antiparasitaire Clinic)	SLP	2015–2020
Dakar	Pikine	Deggo	DEG	2019
St-Louis	Richard Toll	Multiple	RTP	2012–2015

## Data Availability

All the barcode data associated with this study are included in **Supplemental File 1**.
